# A call for action: closing the gap on ethnic disparities in oral cavity cancer care

**DOI:** 10.1038/s41416-025-03186-z

**Published:** 2025-09-10

**Authors:** Nikila Patil, Zainab Lal, Thiagarajan Sridhar, Manish Pareek, Harriet S. Walter

**Affiliations:** 1 Division of Cancer Sciences, School of Medical Sciences, University of Leicester, Leicester, UK; 2 Department of Oncology, University Hospitals of Leicester NHS Trust, Leicester, UK; 3 CRUK/NIHR Leicester Experimental Cancer Medicine Centre, University of Leicester, Leicester, UK; 4 NIHR Leicester Biomedical Research Centre (BRC), University of Leicester, Leicester, UK; 5 Development Centre for Population Health, University of Leicester, Leicester, UK; 6 Division of Public Health and Epidemiology, School of Medical Sciences, University of Leicester, Leicester, UK; 7 Department of Infection and HIV Medicine, University Hospitals of Leicester NHS Trust, Leicester, UK; 8 NIHR Applied Research Collaboration East Midlands, University of Leicester, Leicester, UK

**Keywords:** Oral cancer, Public health, Cancer epidemiology, Health policy

## Abstract

The incidence of oral cavity cancers in the UK is rising. Asian/Asian British ethnic groups and socioeconomically deprived groups are at highest risk with some evidence of worse disease outcomes in South Asian individuals receiving radiotherapy. This variation in incidence and outcomes underscores the urgent need for action.

The incidence of oral cavity cancers (OCC) in the UK is rising. In England, oral cancer rates, including cancers of the oral cavity and oropharynx, have increased by more than 130% since 2000, with mortality also steadily rising [1]. Accumulating evidence suggests that Asian/Asian British ethnic groups and socioeconomically deprived groups are at highest risk. Among Asian/ Asian British individuals, the incidence of OCC is 6.27 per 100,000, compared to 5.96 per 100,000 in White individuals. However, data is limited due to a lack of robust ethnicity data coding and recording [1, 2]. The variation by socioeconomic status is stark with a standardised incidence of 11.13 per 100 000 in the most deprived quintile compared to 6.68 per 100 000 in the least deprived [2]. The drivers of this increased risk are likely to be multifactorial encompassing not only inequities in access to healthcare services but also differences in knowledge, health behaviours and lifestyle risk factors. Biological determinants of disease may also play a role.

The majority of oral cavity cancers are squamous cell carcinomas and the aetiology is strongly linked to risk factors including alcohol consumption and use of tobacco [2]. Alarmingly, around half of oral cavity cancers are diagnosed as Stage IV necessitating more complex and multimodal treatment, with in some situations, palliative treatment being the only option [2].

We recently reported worse disease outcomes in South Asian individuals, defined as self-reporting as Indian, Pakistani and Bangladeshi, receiving radiotherapy for oral cavity cancers in a single centre serving a super-diverse community [3]. We found poorer 2-year disease-free survival in South Asian individuals compared to non-South Asian individuals, 54.6% vs. 73% respectively, even when adjusted for deprivation. Additionally, locoregional recurrence was more frequent in South Asian individuals compared to non-South Asian individuals, 42.5% vs 22.1% respectively, despite similar disease characteristics [3]. A similar study in Canada, also highlighted poorer outcomes in East Indian patients with OCC [4]. It is unknown whether ethnicity, independently, determines patient outcomes or whether there exists an interplay of socioeconomic, cultural and behavioural factors. Variations in genetic, biological and environmental factors between individuals may influence disease trajectory and treatment adherence, tolerability and resultant disease outcome [5]. This has not been fully explored within different ethnic groups.

In the United Kingdom, although smoking remains the most common form of tobacco use across all communities, smokeless tobacco products, whose composition varies significantly, are most commonly used by South Asian individuals, particularly those of Bangladeshi origin [6]. Recreational use of smokeless tobacco and betel nut is strongly linked with variation in the incidence of, and mortality from, oral cavity cancers globally with the highest rates in South-East Asia as well as in South Asian populations in the UK, particularly Indo-Asian immigrants[7–9]. Government action to prioritise competent health messaging and stricter regulation to reduce its use is essential.

Inequitable access to healthcare according to geography and migration status may also influence incidence and outcomes as well as limit dissemination of public health information. With the current crisis in NHS dentistry, a reform of dental services to improve access must be a government priority as this is critical to the early detection and subsequent treatment of oral cancers [10]. Poor oral health is known to increase the risk of oral cancers, thus disproportionately impacting those with limited dental care access [11]. Furthermore, oral health knowledge, attitudes and practices surrounding this are recognised to be inadequate in South Asian migrants [12]. Variation in health seeking behaviours may play a key role in service engagement and applying the Capability, Opportunity, and Motivation – Behaviour (COM-B) model, a behaviour change framework, to understand these can allow the creation of comprehensive and culturally sensitive interventions [13]. Among certain ethnic and migrant groups, a lack of awareness of available services is exacerbated by language barriers which may also hinder the understanding of oral health information disseminated by dental professionals or local authorities. To address this, community outreach and the co-design of information programmes is crucial.

The study of the impact of ethnicity on disease prevalence, incidence and outcome is vital for promoting health equity. A number of OCC cases in the UK are reported as ‘other ethnic group’. This reflects a lack of comprehensive and reliable ethnicity data in UK cancer registries, making it challenging to fully understand and address any disparities that exist [2]. Moreover, migration data would be essential to gaining a deeper understanding of the contribution of environmental, behavioural and socioeconomic determinants on cancer risk and outcomes. In addition, there are significant data gaps on lifestyle risk factors relevant to oral cavity cancers including smokeless tobacco and betel nut chewing. There is also clearly an unmet need to improve diversity and represent those that bear the greatest burden of poor health outcomes in oncology clinical trials [14].

We highlight a disparity in oral cancer incidence and outcomes, underscoring the need for further research and culturally competent interventions. We urge policymakers, researchers and healthcare providers to actively prioritise diversity in clinical trials by developing targeted recruitment strategies, addressing barriers faced by underrepresented communities and collaborating with community health partners. It must be recognised that disparities in cancer care can exist from initial access and diagnosis through treatment and into long term survivorship, so ensuring cultural competence at every stage of cancer care is vital to delivering an equitable healthcare system.
